# Nuclear IHC enumeration: A digital phantom to evaluate the performance of automated algorithms in digital pathology

**DOI:** 10.1371/journal.pone.0196547

**Published:** 2018-05-10

**Authors:** Muhammad Khalid Khan Niazi, Fazly Salleh Abas, Caglar Senaras, Michael Pennell, Berkman Sahiner, Weijie Chen, John Opfer, Robert Hasserjian, Abner Louissaint, Arwa Shana'ah, Gerard Lozanski, Metin N. Gurcan

**Affiliations:** 1 Center for Biomedical Informatics, Wake Forest School of Medicine, Winston-Salem, North Carolina, United States of America; 2 Faculty of Engineering and Technology, Multimedia University, Melaka, Malaysia; 3 Department of Biostatistics, College of Public Health, The Ohio State University, Columbus, Ohio, United States of America; 4 US Food and Drug Administration, Silver Spring, Maryland, United States of America; 5 Department of Psychology, The Ohio State University, Columbus, Ohio, United States of America; 6 Department of Pathology, Harvard Medical School, Boston, Massachusetts, United States of America; 7 Department of Pathology, The Ohio State University, Columbus, Ohio, United States of America; Texas A&M University, UNITED STATES

## Abstract

Automatic and accurate detection of positive and negative nuclei from images of immunostained tissue biopsies is critical to the success of digital pathology. The evaluation of most nuclei detection algorithms relies on manually generated ground truth prepared by pathologists, which is unfortunately time-consuming and suffers from inter-pathologist variability. In this work, we developed a digital immunohistochemistry (IHC) phantom that can be used for evaluating computer algorithms for enumeration of IHC positive cells. Our phantom development consists of two main steps, 1) extraction of the individual as well as nuclei clumps of both positive and negative nuclei from real WSI images, and 2) systematic placement of the extracted nuclei clumps on an image canvas. The resulting images are visually similar to the original tissue images. We created a set of 42 images with different concentrations of positive and negative nuclei. These images were evaluated by four board certified pathologists in the task of estimating the ratio of positive to total number of nuclei. The resulting concordance correlation coefficients (CCC) between the pathologist and the true ratio range from 0.86 to 0.95 (point estimates). The same ratio was also computed by an automated computer algorithm, which yielded a CCC value of 0.99. Reading the phantom data with known ground truth, the human readers show substantial variability and lower average performance than the computer algorithm in terms of CCC. This shows the limitation of using a human reader panel to establish a reference standard for the evaluation of computer algorithms, thereby highlighting the usefulness of the phantom developed in this work. Using our phantom images, we further developed a function that can approximate the true ratio from the area of the positive and negative nuclei, hence avoiding the need to detect individual nuclei. The predicted ratios of 10 held-out images using the function (trained on 32 images) are within ±2.68% of the true ratio. Moreover, we also report the evaluation of a computerized image analysis method on the synthetic tissue dataset.

## I. Introduction

Immunohistochemical (IHC) staining of tissue sections is routinely used in pathology to aid in diagnosis and to characterize malignant tumors in humans and in animals [1–3]. It also plays a vital role in selecting an appropriate systemic therapy for cancer patients [2]. Certain stains are accepted in medical practice as important predictive markers of tumor aggressiveness (Ki67) or tumor type (ER, PR and HER2Neu) and are used to determine the type and intensity of therapy for a given patient [4]. All these markers are used according to specific guidelines where the intensity of stains and the number of positive cells are expressed as percentage of all malignant cells.

Considering the importance of these markers, technical aspects of stains such as tissue fixation, tissue processing and quality of stains are becoming strictly regulated and monitored to assure reproducible results [5]. In the majority of cases, IHC stain interpretation is rendered by a trained pathologist using a light microscope and a manual method, which consists of counting each positively stained cell [5]. As expected, the manual method of enumeration of positive cells suffers from poor reproducibility even in the hands of an expert pathologist [6, 7]. Moreover, due to human reader limitations, true counting of IHC positive and negative cells can only be performed for a limited number of cells [8–11]. Thus, by necessity IHC stains are counted only in small fragments of tumors selected by the pathologist. Such random sampling may be representative of tumors with a homogenous pattern of staining; however, the majority of malignant tumors show a heterogeneous pattern of staining with some areas enriched in positive cells and others with only a few positive cells. Consequently, the counting of IHC positive cells using random sampling of small areas of IHC stained heterogeneous tumors is often biased and does not represent the true nature of the tumor [12]. Oversampling of predominantly positive areas results in overestimation, and oversampling of IHC poor areas results in underestimation of IHC positive cells.

An alternative approach to quantify percentage of positive cells in a high number of cells is estimation based on visual perception of areas without actually counting the positive cells [6]. Due to the limited ability of the human reader to efficiently and reproducibly count hundreds or thousands of cells, the pathologist replaces counting with a more crude method such as estimation (gestalt) [9]. Not surprisingly, such a method introduces substantial bias and variability, and results in poor reproducibility. Considering these difficulties, it is logical that a marked effort is underway to develop computer algorithms to accurately, precisely and reproducibly enumerate IHC stained cells using high resolution images of histological sections. Using different approaches, several competing algorithms have been developed and are currently being tested for clinical use in pathology. Comparison of accuracy and reproducibility between these platforms is based on a reference standard established by human reader interpretation of IHC stains. Paradoxically, all computer method generated results are still compared to results that are generated by a human reader [13]. Using reference standards based on human reader interpretation of images hampers standardization of computer IHC methods mainly due to high inter- and intra-reader variability of humans.

We have developed an *in silico* phantom comprised of both IHC positive and IHC negative (counterstained) cells with a known ground truth, thereby avoiding the need for a reference standard built by human readers. This phantom is generated by our specialized software that uses large collections of high resolution images of different negative and positive cells and generates virtual tissue sections with a known percentage of positive and negative cells. The proportion can be varied from less than 1% to 100% of positive cells. The distribution of positive and negative cells can be adjusted to mimic true histological sections where positive cells can be distributed evenly throughout the tissue or may show focal clustering depending on the intention of the operator. A cluster of cells can be comprised of positive cells with different intensities of staining, different patterns of staining, different cell size with cells showing good separation and cells overlapping (to provide a more challenging task of cell segmentation for computer algorithms).

## II. Methods

**Fig 1:**
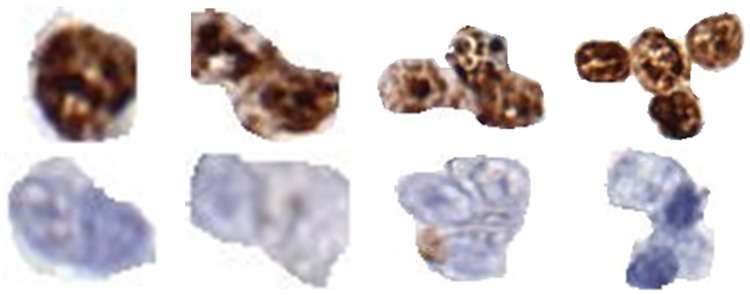
Examples of positive and negative objects used in creation of synthetic images. All objects were cropped from Follicular Lymphoma whole slide images stained for Ki67.

We extracted a group of Ki-67 positive and negative nuclei from images of Ki-67 stained lymphoma biopsies [6, 14]. Each group consists of single nuclei as well as nuclei clumps having two to five nuclei. For simplicity we call these nuclei (single or clumped) “objects.” In total, we have 50 Ki-67 positive objects and 40 Ki-67 negative objects. Fig 1 shows examples of some of these positive and negative objects. The IRB was approved (Study Number: 2007C0069) by the Office of Responsible Research Practices and The Institutional Review Board at the Ohio State University. It is worth mentioning that all images used in this study were fully anonymized.

Let $L_{+}^{i}$ and $L_{-}^{j}$ denote the positive and negative objects, respectively. Here *i* = 1,2,3,⋯50 and *j* = 1,2,3,⋯ 40. To create a synthetic image *I*_*S*_, we perfrom the following steps.

1. Create canvas of 1000x1000 pixels.
2. For each pixel in the canvas, generate a three element vector (with elements corresponding to RGB colors) using a uniformly distributed pseudorandom integer generator between 190 and 250. These values serve as background for the canvas.
3. Select 900 equally spaced points/locations on the canvas. Let (*x*_*a*_,*y*_*b*_) represents the set of 900 locations, where *a* = 1,2,3,⋯30 and *b* = 1,2,3,⋯30.
4. Using a uniformly distributed pseudorandom integer generator, create a set of 900 pairs of random integers between -11 and +11. Let (*Rx*_*a*_,R*y*_*b*_) represent the set of 900 value pairs.
5. Disturb the 900 equally spaced points by adding them with (*Rx*_*a*_,R*y*_*b*_), i.e. (*Px*_*a*_,P*y*_*b*_) = (*Rx*_*a*_, R*y*_*b*_) + (*x*_*a*_, *y*_*b*_). This will perturb the points slightly and will remove the systematic look of computer generated points.
6. To insert G% positive nuclei to the canvas, randomly draw positive objects with replacement from $L_{+}^{i}$ until the total number of nuclei reaches Gx900/100. For instance, to create an image with 40% positive nuclei, we will keep on drawing positive objects until we have drawn a total of 360 nuclei. Then the selected objects are randomly scaled to resemble all different cell sizes in real Ki67 stained tissues.
7. Let us assume that we require N positive objects to create an image with G% positive nuclei. We place these N positive objects in randomly selected N locations from (*Px*_*a*_,P*y*_*b*_).
8. We repeat step 6 and 7 to place negative objects on the canvas. The only difference is that we exclude the N positions already filled by positive objects during extraction of random integers. As a final step, we perform Gaussian filtering on the edges of all positive and negative objects in the canvas.

**Fig 2:**
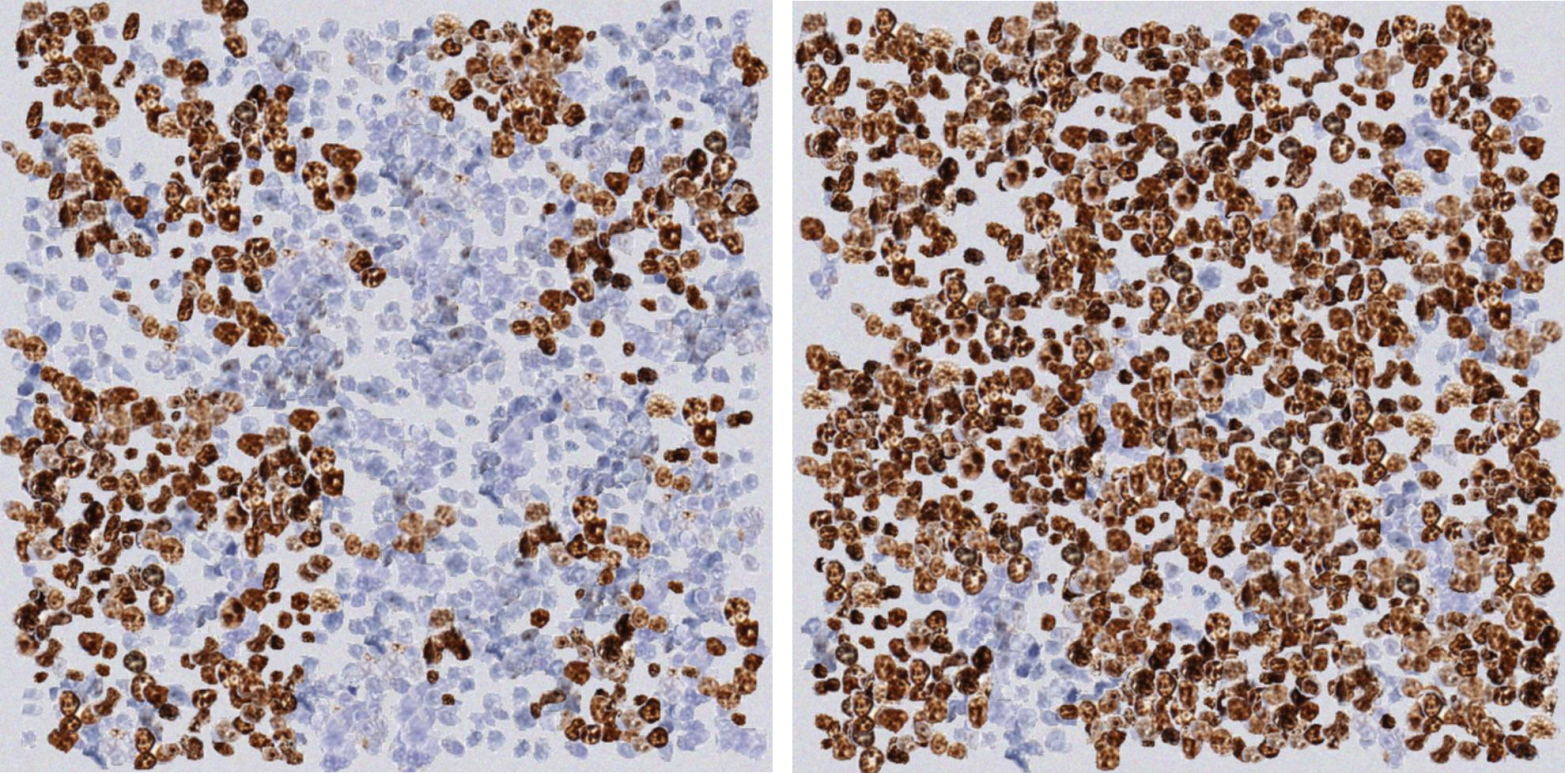
Examples of synthetic images generated by the proposed method. Left) An example image with only a few Ki67 positive nuclei. Right) An example image with higher concentration of Ki67 positive nuclei.

Using this method we created a set of 42 images with different concentrations of positive and negative nuclei. Fig 2 shows an example of two synthetic images created using this method. The 42 synthetic images created using the proposed method were used to investigate the following three questions on Estimation, Mapping, and Validation which are outlined next.

### II.a Estimation

Is a visual estimation of the ratio of positive to total number of nuclei by a group of board certified pathologists consistent with the true ratio? Let ***r***_***n***_ denote true ratio (i.e., the **r**atio of positive to total **n**umber of nuclei during synthetic image generation)

To answer the first question, a total of four board certified pathologists took part in the study. We shared the images through a customized web application, which allowed the pathologists to remotely view the images and provide their visual approximation of ***r***_***n***_, which is denoted as $P_{r_{n}}^{j}$ (j = 1,..4). The pathologists also provided their visual approximation of the following ratio:

$$r_{t}=\frac{Area\;of\;positive\;nuclei}{Area\;of\;the\;image}$$ (1)

### II.b Validation

Can one of our recent nuclei detection algorithms [15] approximate ***r***_***n***_?

In order to evaluate the robustness of the computer based solutions on the artificial dataset, we tested it against one of our recently published nuclei detection algorithms [15]. The algorithm starts by calculating two score maps for blue and brown objects, which correspond to negative and positive nuclei respectively. After calculating the score maps, the algorithm analyses the connected components for different threshold values and finds the optimal threshold value, which maximizes the number of separated nuclei. Finally, the algorithm uses this coarse segmentation result and splits the merged nuclei by detecting elliptical arcs and applying a super pixel method [16].

### II.c Mapping

Can we find a function Ψ(***r***_***a***_) which maps ***r***_***a***_ → ***r***_***n***_, where ***r***_***a***_ corresponds to the **r**atio of the **a**rea of the positive nuclei to the total area of nuclei? The aim is to determine if we can replace the computationally expensive nuclei detection step with the less expensive area estimation.

In order to answer the second question, we divided the 42 synthetic images into 10 subsets (*SS*_1_, *SS*_2_, *SS*_3_,…,*SS*_10_) as shown in Table 1. The third row in Table 1 contains the number of images in each subset. To approximate a function that maps ***r***_***a***_ → ***r***_***n***_, we randomly held out one image from each subset (a total of 10 images which we will use for testing) and computed the following difference for the remaining images (a total of 32 images which we used for training):

$$D=r_{a}-r_{n}$$ (2)

**Table 1:** Division of synthetic images into 10 subsets. Here SS_i_ correspond to the i^th^ subset. The second row contains the ratio of positive to all nuclei within each SS_i_. The third row contains the number of images in each subset.

*SS*_1_	*SS*_2_	*SS*_3_	*SS*_4_	*SS*_5_	*SS*_6_	*SS*_7_	*SS*_8_	*SS*_9_	*SS*_1_
***r***_***n***_ < 0.1	0.1 ≤ ***r***_***n***_ < 0.2	0.2 ≤ ***r***_***n***_ < 0.3	0.3 ≤ ***r***_***n***_ < 0.4	0.4 ≤ ***r***_***n***_ < 0.5	0.5 ≤ ***r***_***n***_ < 0.6	0.6 ≤ ***r***_***n***_ < 0.7	0.7 ≤ ***r***_***n***_ < 0.8	0.8 ≤ ***r***_***n***_ < 0.9	*r*_*n*_ ≥ 0.9
4	4	7	4	5	6	3	3	3	3

## III. Results

For ease of comparison and to avoid redundancy, we combined the results of Subsec*tions II*.*a (Estimation) and II*.*b (Validation)* under Subsection III.a. The results for Subsection II.c are outlined in Subsection III.b.

### III.a Estimation & validation

**Fig 3:**
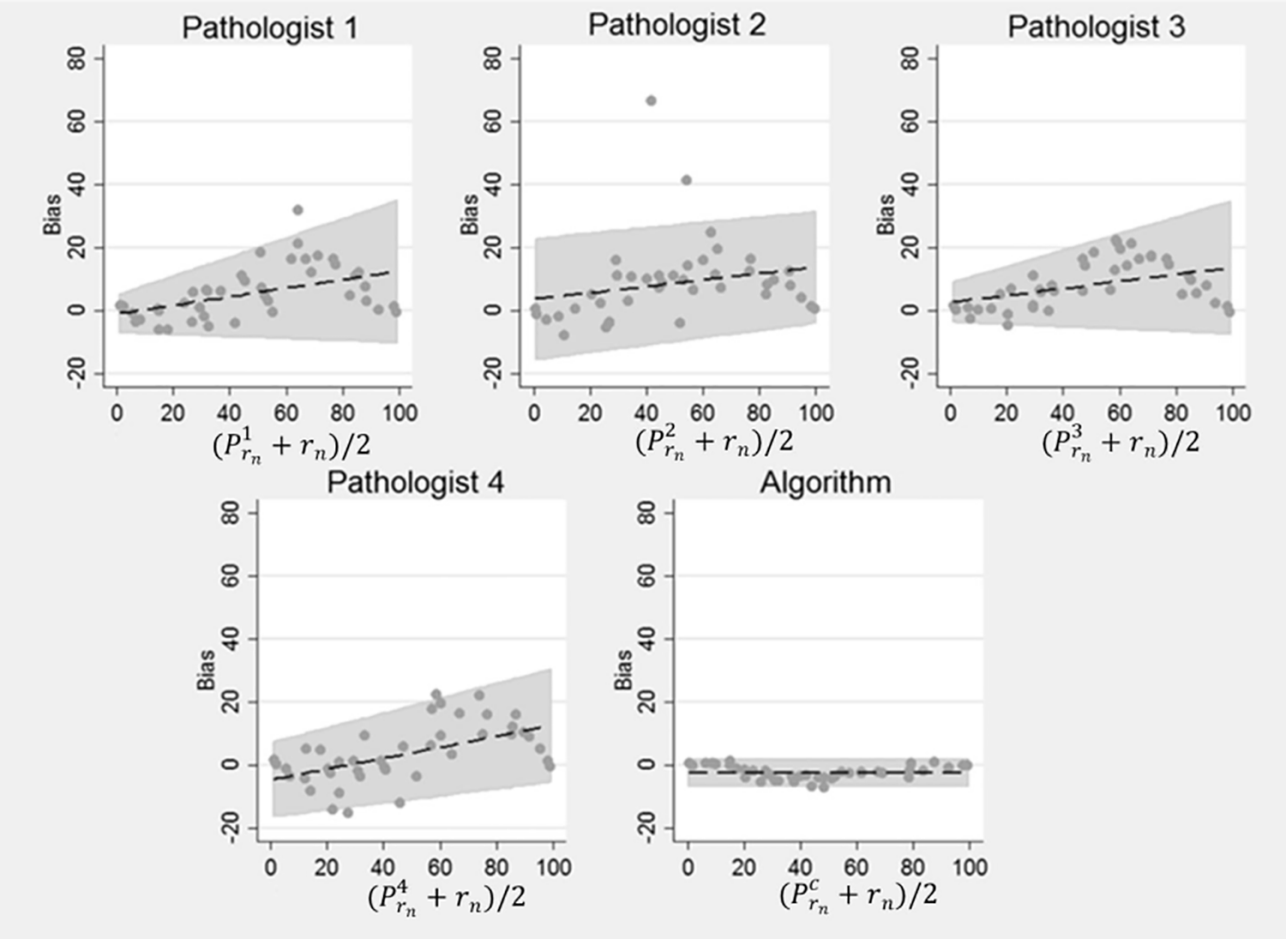
Bland Altman Plots for r_n_. Bias and variability of the pathologists’ estimates of r_n_ increased with the percentage of positive nuclei. However, the bias and variability of the computer’s estimate was relatively smaller than the pathologists’ estimate of r_n_.

**Fig 4:**
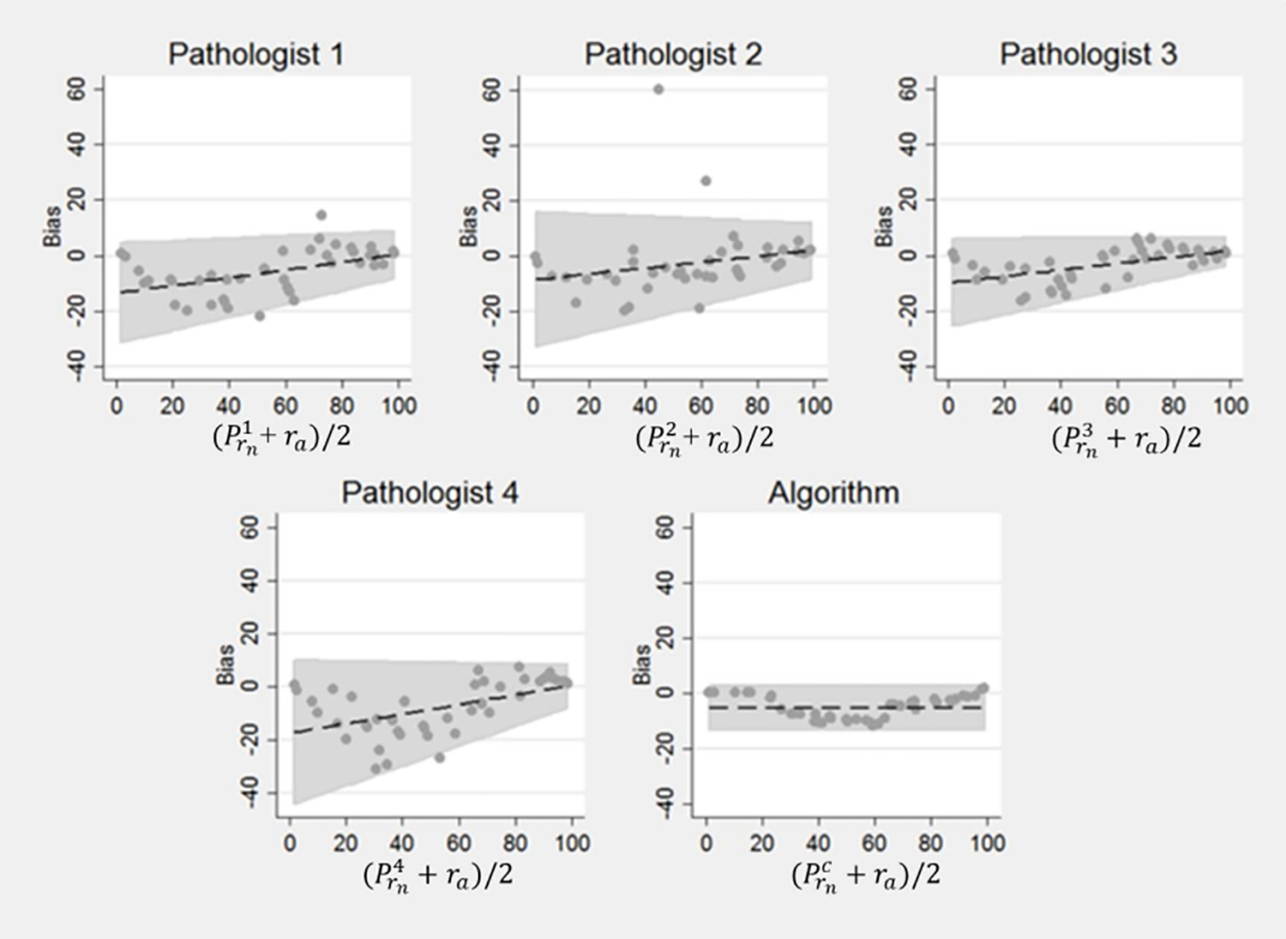
Bland Altman Plots for r_a_. The absolute bias and the variability of the pathologists’ estimates of r_a_ decreased with the increasing percentage of positive nuclei. This shows that for images with higher concentrations of positive nuclei, pathologists’ estimates deviate from accuracy in ways that are not present in the algorithm’s estimates.

**Fig 5:**
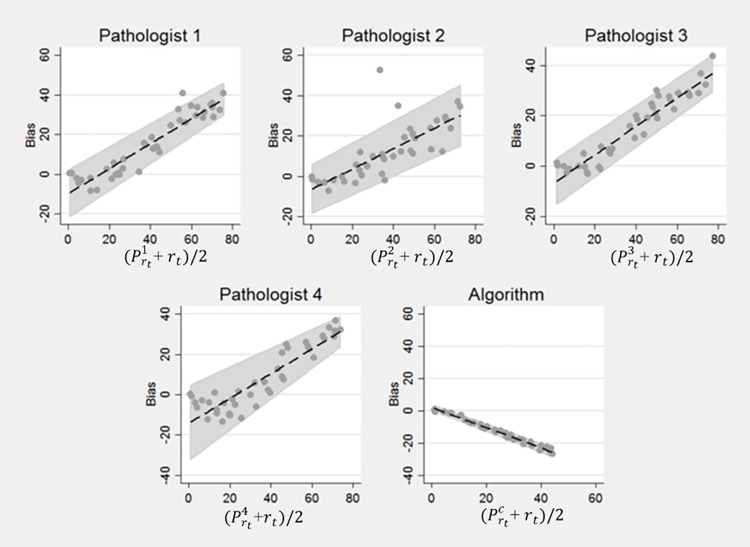
Bland Altman Plots for r_t_. There was a strong increasing linear relationship between pathologist bias in estimating r_t_ and the percentage of positive nuclei. In contrast, there was an almost perfect inverse linear relationship between the bias of the computer’s estimates and percentage of positive nuclei.

The results of our experiment are shown in Tables 2, 3 and 4. Table 2 shows the summary statistics of the pathologists and the computer algorithm in estimating *r*_*n*_ (the ratio of true number of positive to total number of nuclei in an image). The four pathologists’ estimation of *r*_*n*_ is represented by $P_{r_{n}}^{i}$, where i ∈ {1,2,3,4}, while the estimation by the computer algorithm is represented by $P_{r_{n}}^{c}$. Likewise, Table 3 shows the summary statistics of the four pathologists and the computer algorithm in estimating *r*_*t*_ (ratio of the area of the positive nuclei to the total area of the image). Here the total area of the image corresponds to the image size. Similarly, Table 4 shows the summary statistics of the four pathologists and the computer algorithm in estimating *r*_*a*_ (the ratio of the true area of positive nuclei to area covered by all nuclei). It is worth mentioning that we never explicitly asked the pathologists to estimate *r*_*a*_. We used pathologists’ estimates of *r*_*n*_ as their surrogate estimate of *r*_*a*_ in computing the performance metrics in Table 4 and our aim here was to check if the pathologists were unconsciously estimating *r*_*a*_ when requested to estimate *r*_*n*_. CCC and C.I. in all three (Tables 2, 3 and 4) corresponds to Concordance Correlation Coefficient and Confidence Interval, respectively. Similarly Figs 3, 4 and 5 show the results in terms of Bland-Altman plots. These plots enable us to better understand bias and variability among pathologists and the computer algorithms relationship to ground truth.

**Table 2:** Summary statistics of pathologists’ and computer’s estimation of *r*_*n*_ (the ratio of true number of positive to total number of nuclei). Here $P_{r_{n}}^{1},\;P_{r_{n}}^{2},\;P_{r_{n}}^{3},\;P_{r_{n}}^{4},\;P_{r_{n}}^{c}$ denote the estimation of *r*_*n*_ by pathologist 1, pathologist 2, pathologist 3, pathologist 4 and the computer algorithm, respectively.

		$P_{r_{n}}^{1}$	$P_{r_{n}}^{2}$	$P_{r_{n}}^{3}$	$P_{r_{n}}^{4}$	$P_{r_{n}}^{c}$
***r*_n_**	**Mean ± SD**	51.05 ± 31.4	54.2 ± 30.2	53.6 ± 30.6	49.1 ± 32.5	43.1 ± 27.4
	**Bias ± SD**	5.5 ± 8.4	8.6 ± 12.8	8.1 ± 7.5	3.5 ± 9.3	-2.5 ± 2.2
	**CCC**	0.94	0.86	0.93	0.95	0.993
	**(95% C.I.)**	(0.90, 0.97)	(0.77, 0.92)	(0.88, 0.96)	(0.91, 0.97)	(0.987, 0.996)
	**Rank Corr**.	0.98	0.90	0.99	0.97	0.998
	**(95% C.I.)**	(0.96, 0.99)	(0.83, 0.95)	(0.98, 0.99)	(0.94, 0.98)	(0.996, 0.999)
	**Pearson Corr**.	0.97	0.91	0.97	0.97	0.997
	**(95% C.I.)**	(0.94, 0.98)	(0.83, 0.95)	(0.95, 0.99)	(0.94, 0.98)	(0.994, 0.998)

**Table 3:** Summary statistics of pathologists’ and computer’s estimation of *r*_*t*_ (the ratio of the true area of positive nuclei to total image area). Here $P_{r_{t}}^{1},\;P_{r_{t}}^{2},\;P_{r_{t}}^{3},\;P_{r_{t}}^{4},\;P_{r_{t}}^{c}$ represent the estimation of *r*_*t*_ by pathologist 1, pathologist 2, pathologist 3, pathologist 4 and the computer algorithm, respectively. The CCC is lowest for the computer because of the near perfect negative correlation between bias and the number of nuclei (see Fig 5).

		$P_{r_{t}}^{1}$	$P_{r_{t}}^{2}$	$P_{r_{t}}^{3}$	$P_{r_{t}}^{4}$	$P_{t}^{c}$
***r*_t_**	**Mean ± SD**	45.8 ± 30.6	43.8 ± 26.2	46.5 ± 28.5	38.7 ± 29.9	18.0 ± 8.4
	**Bias ± SD**	14.5 ± 15.6	12.5 ± 13.8	15.2 ± 13.1	7.4 ± 15.5	-13.3 ± 7.7
	**CCC**	0.68	0.68	0.69	0.76	0.53
	**(95% C.I.)**	0.57, 0.76)	(0.56, 0.78)	(0.59, 0.77)	(0.68, 0.82)	(0.41, 0.63)
	**Rank Corr**.	0.98	0.90	0.99	0.97	0.992
	**(95% C.I.)**	(0.97, 0.99)	(0.82, 0.94)	(0.97, 0.99)	(0.94, 0.98)	(0.985, 996)
	**Pearson Corr**.	0.97	0.90	0.98	0.95	0.995
	**(95% C.I.)**	(0.94, 0.98)	(0.82, 0.94)	(0.97, 0.99)	(0.91, 0.97)	(0.991, 0.997)

**Table 4:** Summary statistics of pathologists’ and computer’s estimation of *r*_*a*_ (the ratio of the true area of positive nuclei to area covered by all nuclei). Here $P_{r_{n}}^{1},\;P_{r_{n}}^{2},\;P_{r_{n}}^{3},\;P_{r_{n}}^{4},\;P_{r_{a}}^{c}$ represent the estimation of *r*_*a*_ by pathologist 1, pathologist 2, pathologist 3, pathologist 4 and the computer algorithm, respectively. The aim of computing this statistics was to check if pathologists’ are unconsciously computing *r*_*a*_ when asked to approximate *r*_*n*_.

		$P_{r_{n}}^{1}$	$P_{r_{n}}^{2}$	$P_{r_{n}}^{3}$	$P_{r_{n}}^{4}$	$P_{r_{a}}^{c}$
***r*_a_**	**Mean ± SD**	51.05 ± 31.4	54.2 ± 30.2	53.6 ± 30.6	49.1 ± 32.5	51.8 ± 27.8
	**Bias ± SD**	-6.0 ± 8.2	-2.83 ± 12.9	-3.4 ± 6.1	-7.9 ± 10.2	-5.2 ± 4.1
	**CCC**	0.94	0.90	0.97	0.91	0.97
	**(95% C.I.)**	(0.90, 0.97)	(0.82, 0.94)	(0.95, 0.98)	(0.85, 0.94)	(0.95, 0.98)
	**Rank Corr**.	0.98	0.91	0.99	0.97	0.999
	**(95% C.I.)**	(0.96, 0.99)	(0.83, 0.95)	(0.98, 1.00)	(0.95, 0.99)	(0.997, 0.999)
	**Pearson Corr**.	0.97	0.90	0.98	0.96	0.99
	**(95% C.I.)**	(0.95, 0.98)	(0.83, 0.95)	(0.97, 0.99)	(0.92, 0.98)	(0.98, 0.99)

We also performed a separate analysis for each pathologist to allow the relationships to differ by reader. As expected, a pathologist’s estimate of the percentage of positive nuclei can (almost) be perfectly explained by either the true percentage of positive cells *r*_*n*_ and *r*_*a*_ (see Table 5 for details).

**Table 5:** Multiple regression analysis of pathologist rn. Total R^2^ is the proportion of the total variability explained by the true r_n_ and r_a_. R^2^ is the proportion of variability explained by either r_n_ or r_a_ alone. Semi-Partial R^2^ is the additional proportion of total variability explained by the r_n_ or r_a_ beyond the amount explained by the other factor.

		*r*_*n*_		*r*_*a*_	
Pathologist	Total *R*^2^	*R*^2^	Semi-Partial *R*^2^	*R*^2^	Semi-Partial *R*^2^
1	0.9449	0.9421	0.0005	0.9444	0.0027
2	0.8190	0.8164	0.0003	0.8187	0.0026
3	0.9699	0.9692	0.0023	0.9677	0.0007
4	0.9333	0.9146	0.0040	0.9293	0.0187

### III.b Mapping

**Fig 6:**
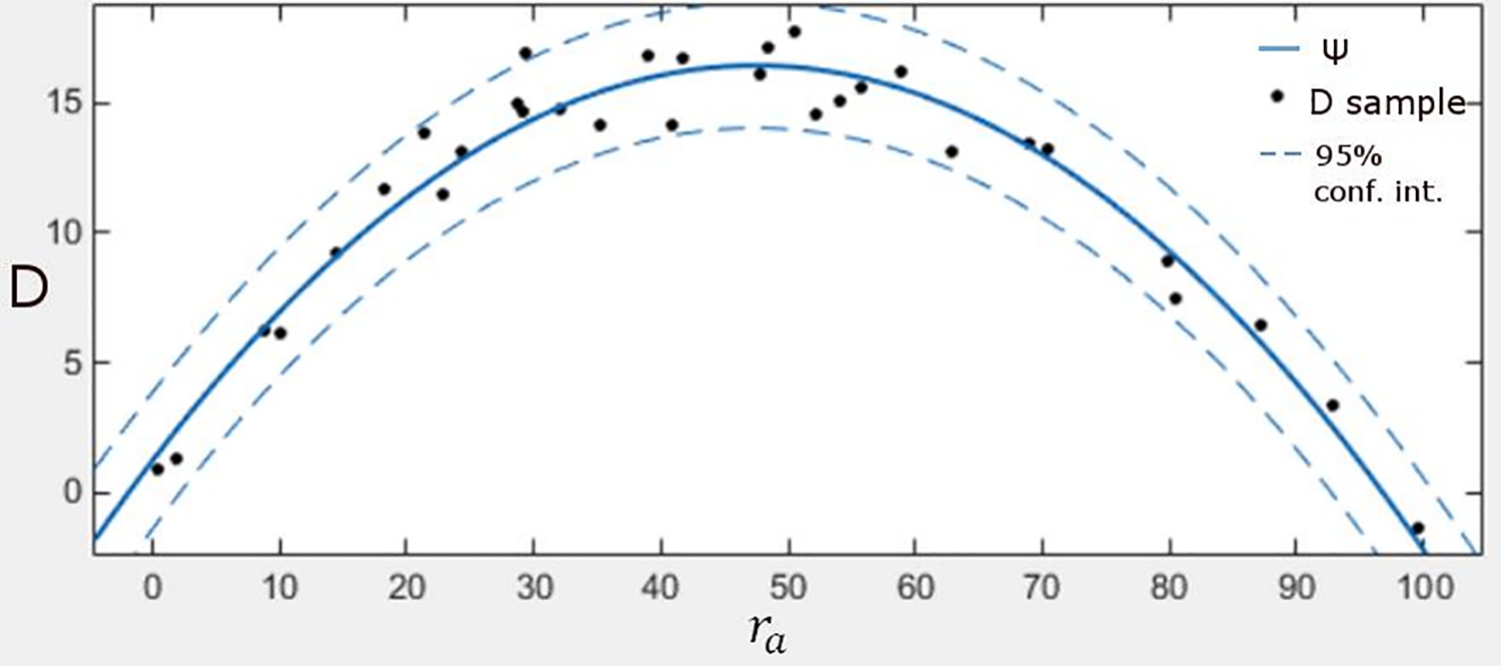
The plot shows a function which facilitates the mapping of *r*_*a*_ → *r*_*n*_. The horizontal axis corresponds to different values of *r*_*a*_ while the vertical axis represents D, i.e. error. Each individual dot represents the error between *r*_*a*_ and *r*_*n*_ for the training images. The solid line corresponds to the mapping function *Ψ* which facilitates the mapping of *r*_*a*_ to *r*_*n*_ while the dotted lines represent the confidence interval.

Table 6 shows the approximation of r_n_ for the 10 test images. Here *$\hat{r_{n}}$* represents the approximation of r_n_ through Eq 5. Table 6 was generated using leave-one-out cross validation methodology. Fig 6 shows a plot of *D* (as dots) for 32 training images along with a function that approximates these values. The horizontal axis in Fig 6 corresponds to r_a_ while the vertical axis represents D. The objective is to find a function that maps *r*_*a*_ to ***r***_**n**_. The dotted lines in Fig 6 correspond to the 95% confidence interval. The continuous solid line represents a function that approximates *D* using the following 2^nd^ degree polynomial:

$$D\approx \Psi (r_{a})=a\times ({r_{a})}^{2}+b\times r_{a}+c,$$ (3)

wherea=−0.007,b=0.64,c=1.26

**Table 6:** Approximation of r_n_ for the 10 test images. Here $\hat{r_{n}}$ represents the approximation of r_n_ through Eq 5.

Test image	*r*_*a*_*(%)*	*r*_*n*_*(%)*	$\hat{r_{n}}$(%)
1	61.99	44.11	46.98
2	49.27	35.18	32.86
3	97.47	97.82	97.89
4	29.92	18.21	15.56
5	42.92	28.68	26.63
6	75.11	58.87	63.83
7	43.82	29.13	27.48
8	55.72	38.95	39.75
9	14.92	8.70	5.62
10	63.95	47.81	49.35
**Standard deviation of the difference between r**_**n**_ **and $\hat{r_{n}}$**			**±2.68%**

We determined the coefficients *a*,*b*,*c* by solving the following minimizing problem:

$$\underset{x}{\text{min}}{∥D(x)-r_{a}∥}_{2}$$ (4)

Here *x* corresponds to the vector of coefficients *a*,*b*,*c*, i.e., $x=\left[\begin{array}{c} a\\ b\\ c \end{array}\right]$.

This means that ***r***_**n**_ can be approximated by substituting *D* with Ψ(r_a_) in Eq 2, i.e.

$$\Psi (r_{a})\approx D=r_{a}-r_{n}$$ (5)

$$r_{n}\approx r_{a}-\Psi (r_{a})$$ (6)

## IV. Discussion and conclusions

The bias and agreement of the pathologists relative to the ground truth and those of our computer algorithm [15] are described in Tables 2–4 and the Bland-Altman Plots in Figs 3, 4 and 5. The level of agreement is captured in Tables 2–4 by the value of the CCC. Generally, the larger values of CCC indicate better reproducibility between two variables. Pathologist bias and agreement with r_n_ and r_a_ were less biased and in better agreement than their estimates of r_t_. However, variability and absolute bias of the pathologists’ estimates of ***r***_**n**_ increased as the number of positive cells increased (Fig 3) while the opposite was true when pathologist estimates were compared to ***r***_a_ (Fig 4). There was a strong increasing linear relationship between pathologist bias in estimating ***r***_t_ and the percentage of positive cells (Fig 5). The computer algorithm exhibited low bias and almost perfect agreement with ***r***_n_ and ***r***_a_, but there was an almost perfect decreasing linear relationship between the computer algorithm bias and the percentage of positive nuclei. In contrast to the pathologist estimates, the variability of the computer’s estimates was smaller and was independent of the number of positive nuclei. From the summarized statistics in Table 2, it is clear that visual estimations by pathologists are not a reliable means to approximate ***r***_n_.

Table 6 suggests that Ψ(***r***_a_) provides a reasonable approximation of ***r***_n_. The standard deviation of the differences between approximated values of ***r***_n_ and true ***r***_n_ is small (2.68%). State of the art nuclei detection algorithms result in around 5–10% false detection rate [13, 17–19]. So, from that perspective, this is an extremely useful result as it 1) has the potential to replace computationally expensive nuclei detection algorithm with inexpensive area estimation, and 2) promises to produce more accurate or at least as good results as any state of the art nuclei detection method. But it still requires extensive validation and testing on a real dataset. At the moment, the synthetic nature of our images allowed us to compute ***r***_a_ by simply counting the number of positive and negative nuclei. However, the generalization of the mapping function to real data requires a selection of an automatic segmentation algorithm to approximate ***r***_a_.

Accurate IHC quantification is essential; improper quantification of IHC stains can deny a patient an important therapy option or result in unnecessary treatment. Unfortunately, IHC quantification can vary significantly between readers, as well as within readers [20, 21], for reasons that are neither fully understood nor easily addressed. Both quality assurance and pathologist training programs designed to standardize IHC stain interpretation suffer from lack of absolute standards with known ground-truth. Our Phantom generator allows creation of unlimited quantities and varieties of digital images that can be used for quality assurance programs, training of pathology residents and development of computer algorithms that will help to solve problem of poor reproducibility of IHC stains interpretation and quantification in clinical practice of pathology.

IHC stains are used to characterize the repertoire of antigens (mostly proteins) that are expressed by malignant cells. Certain antigens are very specific and some are less specific but sensitive in determination of cell types and properties. For example most of carcinoma cells will express various keratin molecules and appear negative for common leukocyte antigen CD45. On the other hand, most leukemia/lymphoma cells will be negative for keratin and will be positive for CD45 antigen. Similarly different antigens are expressed by sarcoma cells. These antigens are negative in carcinoma and lymphoma cells while antigens expressed by carcinoma and lymphoma cells are negative in sarcoma cells. IHC stains are essential in detecting these differences and thus very useful in differentiating between carcinoma, lymphoma and sarcoma cells.

Computationally, this finding suggests savings on computation time, considering that most nuclei detection algorithms [19, 22] take hours (if not days) to approximate the number of nuclei to facilitate the computation of r_n_. This mapping function if used in conjunction with the method in [6, 14] has the potential to reduce this time to minutes. However, this function (*i*.*e*.*g* Ψ(*A*)) is specific to images of Ki-67 stained follicular lymphoma biopsies. Its generalization to other tumors may require re-estimation of Ψ(*A*). It is important to perform a detailed analysis of Fig 6 to understand the quadratic behavior of the mapping function, Ψ. When there are only a few positive nuclei, it results in the smallest error. This is evident from the y-axis of the plot in Fig 6; the errors are small when there are few positive nuclei and increase as the percentage of positive nuclei approaches 50%. The function starts to decrease in a quadratic fashion after this point until it reaches a point where the image only contains positive nuclei. So, it is essentially a function of homogeneity; it results in least amount of error when the image contains one type of nuclei. The function increases with the increase in heterogeneity.

By generating a series of virtual tissues (phantoms) with different proportions and distribution of positive and negative cells, the exact number of positive and negative cells is known, therefore, it can be used as a gold standard for the following purposes:

- An IHC standard for the industry to test computer algorithms for enumeration of IHC stained cells.
- IHC standards for pathology quality assurance programs such as those administered by 9AP and similar organizations for programs used to standardize breast cancer pathology, lung cancer pathology, lymphoma pathology, etc.
- Using these Phantom standards with different proportions of positive and negative cells, one can print hundreds of high resolution slides on glass that can be used for testing with light microscopy and for testing of high resolution slide scanners.
- By further expanding this model, one can generate 3D phantoms of tissue with different proportions of negative and positive cells. Using 3D printers, one can print artificial tissue using a cartridge of collagen, or other matrix, with a positively and negatively stained suspension of cells. These 3D printed tissues could be used to standardize histology processing of tissue fixation and tissue cutting. Using 3D printers with cartridges of collagen or other Matrix and unstained cells with a known immunophenotype, one can generate (print) 3D artificial tissues that can be used as a standard for tissue fixation, tissue processing, IHC staining using different IHC platforms, image acquisition, and image IHC analysis.

The main objective of this study was to provide a reliable approach to generate ground truth in the study and analysis of IHC stained tissues. In most studies of IHC stained images, obtaining the ground truth is a major challenge due to various factors such as limited availability of experts to perform annotations, inter- and intra reader-variability and limited datasets. Often, the ground truth is either a generalization of reader consensus or the average of readings among multiple readers which may be biased, hence disputed. This method allows generating virtual images mimicking IHC stained tissue with exact proportions of positive and negative nuclei with controlled distribution of stained nuclei. These virtual tissue images can be used as the gold standard for IHC quantification for both computer based image analysis methods and quality assurance programs for manual evaluation of IHC stained tissue by pathologists. It creates virtual IHC stained sections with a known percentage of positive and negative cells and eliminates bias that is associated with IHC standards where the ground truth is based on manual counting of positive nuclei by pathologists. The study also showed that it is possible to approximate the ratio of positive to total number of nuclei from areas of positive and negative nuclei. The study also evaluated the performance of one of our recently developed nuclei detection algorithms [15].

In the future, we are planning on generating whole slide images which contain structures like blood vessels and other types of nuclei. We are also planning on extending it to other disease types including lymphoma, different types of breast cancer, lung cancer, prostate cancer, etc. For each tumor type phantom tissue can be further complicated by introduction of other objects such as lymphocytes, histiocytes, blood vessels, nerve bundles, fibrotic fibers, and artifacts such as hemorrhage of necrosis.

The current approach lacks the ability to generate complex structures like ducts and lobules in breast. Moreover, it is relatively hard to generate a particular distribution of nuclei with the current design. Similarly, it requires a bit of tuning if the objective is to orient nuclei in a certain direction.

## Supporting information

S1 File Interactive Image Compression for Big Data Image Analysis: Application to Hotspot Detection in breast cancer. (PDF)
