# Supplementary material for: Nuclear IHC enumeration: A digital phantom to evaluate the performance of automated algorithms in digital pathology
Source: PLoS One. 2018 May 10;13(5):e0196547. doi: 10.1371/journal.pone.0196547 (PMC5944932; doi:10.1371/journal.pone.0196547)
Supplement: S1 File — (PDF) [file pone.0196547.s001.pdf]

Interactive Image Compression for Big Data Image Analysis: Application to Hotspot Detection in Breast Cancer

M. Khalid Khan Niazi[1], Y Lin[2], F. Liu[2], A. Ashok[2], M. W. Marcellin[2], G. Tozbikian[3], M. N. Gurcan[1], A. Bilgin[2]

[1]Department of Biomedical Informatics, The Ohio State University, Columbus, Ohio, USA

[2]University of Arizona, Tucson, AZ, USA

[3]Department of Pathology, The Ohio State University, Columbus, Ohio, USA

Corresponding Author: M. Khalid Khan Niazi,

Email: Muhammad.niazi@osumc.edu

Phone: 1-614-699-9752

Fax: 1-614-688-6600

Keywords: Compression, pathology images, JPIP, Ki-67, hotspot detection, Alpha shapes

ABSTRACT

In this paper, we propose an interactive image compression framework to address the needs of Big Data image analysis in digital pathology. Big Data image analytics require analysis of large databases of high-resolution images using distributed storage and computing resources along with transmission of large amounts of data between the storage and computing nodes that can create a major processing bottleneck. The proposed image compression framework is based on the JPEG2000 Interactive Protocol and aims to minimize the amount of data transfer between the storage and computing nodes as well as to considerably reduce the computational demands of the decompression engine. The proposed framework was integrated into hotspot detection from images of breast biopsies, yielding considerable reduction of data and computing requirements.

I. INTRODUCTION

Modern imaging techniques generate very large images and the wide-spread utilization of image data in many areas has created significant challenges associated with processing, analysis, and management of these large datasets. The need to handle large volumes of image data is pervasive today in nearly every industry, government, and institutional sector. For example, in digital pathology, a single digital whole slide image produces a file ranging in size from 2 to 60 gigabytes (GBs). When an entire case is considered (typically between 4 to 30 different slides), the raw data size can exceed 100 GB. Considering the daily slide volumes of a typical academic pathology department, the volume of data generated by a fully digital pathology practice can be enormous.

It is important to point out that the information present in medical images has immediate as well as long-term relevance: The pathology images associated with a case have immediate diagnostic relevance for that case. However, some information in these images becomes relevant only when it is considered as part of a large cohort. Subtle characteristics of disease can only be identified when a large set of images are analyzed collectively. Therefore, there

has been growing interest in Big Data analytics in information sciences over the last few years [1]. While extraction and quantification of relevant [2] and task-specific information [3] from images has long been an active area of research within the image processing community, the emergence of Big Data poses additional challenges particularly in processing resources/speed and scalability.

One of the most prominent Big Data challenges in histopathological image processing is how to transmit, store, and, most importantly, manage this large amount of data efficiently. Conventional image compression methods were designed to act as an input-output filter used for compression enabling access to the image at a single image quality, size, resolution, and spatial extent that was envisioned at the time of compression [4]. However, these conventional methods are impractical for Big Data image analytics. Many Big Data image analytics applications employ *distributed storage* and *computing*, and *transmission* of large amounts of data between the storage and computing nodes creates a major processing bottleneck. Furthermore, Big Data databases are often mined for different tasks and the image quality, resolution, and spatial extent relevant for different tasks can be vastly different [5]. In this work, we propose an interactive image compression framework that acts as part of a Big Data histopathological image processing system providing efficient and scalable interaction with histopathology images. The proposed framework is based on the JPEG2000 image compression standard [6] and the JPEG2000 Interactive Protocol (JPIP) [7]. Through the use of an example application, we illustrate that when image compression is tightly integrated with the image processing methods, both compression and computational efficiency can be significantly improved. So, the aim of this study is to develop and validate a novel image compression paradigm where information most relevant to the task at hand is stored and transmitted preferentially.

II. SUBJECTS AND METHODS

The JPEG2000 image compression standard was designed with the central theme of scalability. In addition to resolution and quality scalability, JPEG2000 provides spatial and image component accessibility. This rich family of scalability features enables JPEG2000 code-streams to be easily parsed to extract subsets of compressed data that represent a desired region of interest with a desired set of color components, at a desired resolution and with a selected level of quality. The JPIP protocol [7] builds on this strong foundation to allow interactive access to JPEG2000 compressed images over networks. The block diagram in Figure 1 illustrates the basic architecture of a JPIP server and client. **[Figure 1.]**

As suggested in the figure, the client sends request to the server about its desired region, resolution, and color component of interest and the server responds by transmitting the compressed data that is necessary to obtain an image with the desired attributes. While the JPIP framework has been shown to be useful for remote browsing of very large images, it can also provide the essential ingredients for image processing applications that employ distributed storage and computing. The JPIP client can be integrated within the image processing method and communicate the current region, resolution, and color components of interest to the server which, in turn, transmits the corresponding compressed data to the client. A region decompressor can then decompress this compressed data and return it to the image processing method. Note that this framework not only minimizes the amount of data transfer between the storage and computing nodes but also minimizes the computational resources consumed by the compression engine since only data required by the image processing method is transmitted and decompressed. Using the Kakadu Software Development Kit [8], we have created a MATLAB® (Mathworks, Natick, Massachusetts) interface which allows seamless integration of a JPIP client with image processing methods. Using this interface, we have implemented a distributed image analysis method. This method is presented in the next section.

1. Application to Hot-Spot Detection in Ki-67 Stained Breast Tissue

Ki-67 is a nuclear protein expressed exclusively during the active cell cycle phases with no expression in quiescent cells [9]. Its presence appears to be necessary for cell proliferation, although its exact function is unclear [10]. In breast cancer, Ki67 has shown promise as an independent prognostic marker and as a predictive marker of responsiveness or resistance to chemotherapy or hormone therapy [11]. According to the published recommendations of the Breast Cancer working group [12], Ki-67 score or index is defined as the percentage of positively stained cells within the total number of malignant cells scored. In Ki-67 scoring, hotspots are generally defined as areas in which Ki-67 staining is most prevalent; or those areas with the highest number of positively staining nuclei within the invasive component.

From image analysis perspective, hotspot detection can be considered as a density approximation problem. In the past, hotspot detection was partially addressed by a few studies [13-15]. Some of these earlier methods [14, 15] were only tested on small region-of-interest images and their extension to whole slide images is not trivial due to computational challenges. In this work we present an efficient method that approximates the hotspots from a whole slide image within reasonable time using our proposed compression frame-work. The hotspot detection component of this framework is generalization of [16, 17] to whole slide images.

Ki-67 positive cells manifest themselves as brown hue cells in images of breast tissues. The large variations in specimen preparation, staining, and imaging as well as true biological heterogeneity of breast tissue often results in variable brown intensities in Ki-67 stained images [17]. These variations affect the segmentation accuracy of Ki-67 nuclei. We performed segmentation of breast tissue images using an efficient version of [17]. This method exploits the intrinsic properties of CIE $L^*a^*b^*$ color space to translate this complex problem into an automatic entropy based thresholding problem. The method in [17] consists of three main components; *clustering of RGB color pixels into three clusters based on cluster*

centroids, color space transformation in the CIE $L^*a^*b^*$ color space, and entropy thresholding to segment the Ki-67 positive nuclei. Computationally, clustering is the most expensive component among the three. The absence of a closed form solution for clustering and the need for iterative refinement turns [17] into a computational bottleneck. Moreover, the method was originally designed for region of interest (ROI) images with an assumption that each ROI image has some Ki-67 positive nuclei. However, its block-by-block application to a whole slide image usually contains blocks where Ki-67 positive nuclei are completely absent. In those situations, the method erroneously starts to consider negative nuclei as Ki-67 positive nuclei. To reduce the computational complexity and the number of false positives, we propose an efficient version of [17] which extends its application to whole slide images. The efficient version consists of three main steps; automatic ROI selection from a whole slide image, extraction of parameters from the selected ROI, and application of [17] to whole slide images with the extracted parameters.

1) Automatic Selection of an ROI from a whole slide image

The aim of this step is to automatically select an ROI image with some Ki-67 positive nuclei. To accomplish this, we manually cropped 25 ROI images with different concentration of Ki-67 positive nuclei from 25 different whole slide images. The size of these ROI images varies between 1K×0.5K and 9K×5K. Nine out of 25 whole slides images were acquired at Cleveland Clinic while the rest were acquired at The Ohio State University. From the 25 manually cropped ROI, we extracted: cluster centroids, the color transformation matrices, and the thresholds resulting from entropy thresholding. Figure 2 shows the resulting cluster centroids as piecewise linear functions. Each function consist of nine points, the first three points correspond to first cluster centroid, the next three points represent the second cluster centroid, and the last three points represent the third cluster centroid. Although all ROI images contain Ki-67 positive nuclei, there exists a huge variation in cluster centroids across images. However, the cluster centroids seems to differ less if the ROI images (containing Ki-67 nuclei) are selected from within the same slide. This prompted us to automatically find an

ROI from the whole slide image and apply the resulting cluster centroids to whole slide image.

To accomplish this we computed the average centroid matrix, $\bar{C}$ as:

$$\bar{C} = \sum_{n=1}^{25} \frac{C_n}{25} = \begin{bmatrix} 88.4 & 176.1 & 215.9 \\ 68.3 & 184.2 & 218.9 \\ 64.2 & 203.3 & 227.5 \end{bmatrix}$$

Here C_n is the cluster centroid matrix for ROI image n. The columns of the resulting matrix correspond to the cluster centroid. It is worth mentioning that $\bar{C}$ is just an approximation. The true cluter centroids will be extracted after automatic selection of a ROI.

For visulization and other practical reasons, most whole slides images are stored in multi-page format. Our 25 whole slide images in the training dataset are also stored in multipage Tiff format. We extracted a 2.5x magnified image from the multipage Tiff file. The resulting images at 2.5x magnification for our training datset are nearly 6K×6K in size. We selected 2.5x magnification as the number of Ki-67 nuclei extracted are relatively close to what we get at 40x magnification (highest magnification). The number of Ki-67 positive nuclei tend to drop drastically in images with less than 2.5x magnification. For each 2.5x whole slide image in the training set, we grouped RGB color pixels based on its elucdian distance from $\bar{C}$.

[Figure 2.]

Apart from the computation of the color transformation matrix, rest of the steps are the same as explained in [17]. Figure 3 shows the resulting color transformation matrices as piecewise linear function for 25 ROI images. The Eigen vectors in each color transformation matrix are first appended next to each other, hence resulting in a 9 element vector as shown in Figure 3. Interestingly, the color transformation matrix (Figure 3) seems nearly identical for the 25 ROI images. This allowed us to use an average of this transformation matrix, $\overline{CT}$ as a color transformation matrix for whole slide images. So, instead of re-computing the color transformation for each block of the whole slide image, we used the same transformation matrix for all of the whole slide images.

$$\overline{CT} = \begin{bmatrix} 0.996 & -0.031 & 0.043 \\ -0.040 & 0.195 & 0.978 \\ 0.0395 & 0.975 & -0.194 \end{bmatrix}$$

Figure 4 shows the threshold values in terms of a graph. The graph reveals that the threshold values changes considerably in each image. For this reason, we computed individual thresholds for each block in a whole slide image.

[Figure 3.]

[Figure 4.]

In summary, while $\bar{C}$ and $\overline{CT}$ were kept unchanged for all 2.5x images, we computed the individual threshold using entropy thresholding. The pre-computation of $\bar{C}$ and $\overline{CT}$ bring considerable computational savings as these do need to be iteratively refined for clustering.

The next step in automatic selection of an ROI is to segment all 2.5x images with precomputed $\bar{C}$ and $\overline{CT}$ and entropy thresholding. Once segmented, the images are reduced to 0.25x magnification using bilinear interpolation. The resulting images are again converted into binary images by setting all non-zero elements to 1. The use of bilinear interpolation followed by setting of non-zero elements to 1 ensures that any potential nuclei are not lost as a result of down sampling. The next step is to group the location of the resulting non-zero pixels into seven clusters. One may opt for different number of clusters than 7. Our choice was mainly driven by computational efficiency as it resulted in a large enough ROI region to reliably extract true cluster centroids. The automatic selection of an ROI takes nearly 8 seconds per image. As a last step, we use the convex shape of the cluster with the minimum point to centroid distance as an automatically selected ROI.

Once a ROI is automatically selected, we compute the true cluster centroids according to the method in [17]. For accurate segmentation of the whole slide images, we used true cluster centroids, $\overline{CT}$, and entropy thresholding at (4x) lower-resolution images. For images of size larger than 90K×90K pixels, lower resolution images (8x) were used.

In [16], we used an automated method to detect individual nuclei from nuclei clumps at 40x magnification. However, this method is computationally demanding. For computational efficiency, we benefit from relatively uniform size of Ki-67 positive nuclei to approximate the number of nuclei within nuclei clumps. During training, average nucleus size was manually measured and it was used during testing to approximate the number of nuclei within clusters of nuclei.

Once the nuclei centroids are approximated, we used a modified version of α-shape maps [16] to generate a heat-map from nuclei centroids. The α-shape was computed from the k-nearest centroids of the ith centroid. α-shape is a generalization of convex hull to non-convex shapes and attempts to define the shape of a finite set of point in the space. Each α-shape is a binary image where the points inside the α-shape are assigned the value of 1. We further compute the weighted map by using the equation:

$$w\alpha^m = \sum_{i=1}^{n} \left\{ \alpha_i \times S_{C_i}^k \times \Lambda_i\left(\alpha_i\left(S_{C_i}^k\right)\right)\right\}$$

Here, Λ_i is the area function which computes the area of the ith α-shape. To compute the heat map, H^m, we compute:

$$H^m = \frac{\alpha^m}{w\alpha^m}$$

However, computing α-shapes in a large size image is computationally challenging. As we are only utilizing the nuclei centroids (locations), we can afford to use a much lower resolution image to generate heat-maps without losing much information. Dividing the location of nuclei centroids by a factor of *d* directly corresponds to *d*-times lower resolution of resulting heat-map. However, dividing the location by a factor of *d* will not result in loss of any nuclei but will only bring them closer in proximity. In our experiments, we set *d* = 10 as it results in least number of overlapping nuclei. After generating the heat maps, the heat map is segmented using fast marching [18]. We used gray-scale intensity difference as weights for image pixels. The top 2% (highest values in the heat-map) were set to initialize the fast

marching. Once again, this step was also performed at the same resolution as the heat-maps.

III. RESULTS

This hotspot detection method was implemented in MATLAB and integrated with the proposed client-server framework. A total of 50 images of breast tissue scanned at 40× magnification with ScanScope^{TM} (Aperio, Vista CA) were used in this study. The images ranged between 2.2 gigapixels to 53 gigapixels in size. An expert pathologist manually annotated the tumor as well as the hotspots in all of the images. We selected 25 images for training and 25 for testing. All processing was performed within tumor regions which were outlined by the pathologist. The hotspots detected by the proposed method were evaluated against the hotspots outlined by the pathologists. For 5 images, we detected more hotspots then the pathologist. Those images were then re-evaluated by the pathologists. After the re-evaluation, the pathologists agreed with the extra hotspots detected by the proposed method.

Three different approaches for image transfer were evaluated within the proposed framework and the results of the experiments on seven images are provided in Table 1. The Table was only limited to seven images due to space constrains. The first approach (denoted as "Full Tissue Image" in the table) corresponds to a "conventional approach" where the entire *compressed* codestream is transferred to the computing node. In this case, the average effective compression ratio (ECR) calculated as the ratio of the number of bytes used to represent the uncompressed image to the number of bytes transferred over the network is roughly 15 which correspond to the compression ratio of the original JPEG2000 code-streams stored on the server. In addition, the runtimes for decompression of the transferred data are also tabulated in Table 1. It is easy to see that the decompression runtimes in this case are high and considerably exceed the runtime of the hotspot detection method. The second approach (denoted as "Full ROI" in the table) corresponds to an intermediate

integration of the hotspot detection method with the proposed framework. In this case, the tumor region is determined and a minimum enclosing rectangular region of interest around the tumor region is requested from the server for further processing. The average ECR in this case is 127, roughly an order of magnitude higher than the ECR of the conventional method. Correspondingly, there is a considerable decrease in the decompression runtimes as well. Finally, the third approach (denoted as ”Only ROI” in the table) represents a much tighter integration of the client software with the hotspot detection method where only the tumor region is requested from the server (instead of the enclosing rectangular region). The average ECR in this case is 222 and the decompression runtimes are on average 15 times faster than the conventional approach.

[Table 1.]

IV. DISCUSSION & CONCLUSIONS

Clinicians and researchers in histopathology are increasingly generating and using whole slide images. The slide volumes of a typical academic pathology department require round-the-clock operation of multiple scanners which can be loaded with hundreds of slides and can scan continuously [19]. Thus, the volume of data that is expected to be generated by a fully digital pathology practice is enormous. In addition to tremendous storage requirements, the large data sizes also present challenges for rapid and interactive access to image data. High performance image rendering with low-lag times are critical to match or exceed the current productivity levels of pathologists using the light microscope. While there is an increased interest in the use of quantitative image analysis algorithms for disease detection, diagnosis, and prognosis prediction to complement the opinion of the pathologist, these algorithms require efficient access to image data. The practice of pathology in the era of Big Data requires development of advanced data compression methods. The current manuscript is geared towards creating an integrated computational framework for image compression

that allows us to efficiently store, process, and provide rapid access to the high quality images. In this regards, our contribution is twofold;

- We presented a task specific image compression framework which provides the user with the flexibility to perform *context aware image compression*. This enabled us to use a variable compression rate within the same image. As a consequence, the proposed framework minimizes the data transfer between storage and computing nodes and significantly reduces the computational resources consumed by the decompression engine.. For instance, the variable nature of our framework allows for higher compression ratios in areas outside of the tumor and relatively lower compression ratios within the tumor region. This is currently not possible with the existing compression methodologies.
- We presented an *efficient* method to detect hotspots from whole slide images of breast tissues. On average it took 88.6 seconds (standard deviation of 36.3 seconds) to detect hotspots from whole slide images. This processing time does not include the time required to read/transfer the image.

Natural directions for future research include exploration of the impact of quality scalability features with various image processing methods as well as incorporation of task-based image quality metrics into the proposed framework.

Acknowledgement:

This work was supported in part by the National Institutes of Health (NCI 1U01CA198945-01).

REFERENCES

[1] C. P. Chen and C.-Y. Zhang, "Data-intensive applications, challenges, techniques and technologies: A survey on Big Data," *Information Sciences,* vol. 275, pp. 314-347, 2014.

[2] H. H. Barrett, J. Yao, J. P. Rolland, and K. J. Myers, "Model observers for assessment of image quality," *Proceedings of the National Academy of Sciences,* vol. 90, pp. 9758-9765, 1993.

[3] M. A. Neifeld, A. Ashok, and P. K. Baheti, "Task-specific information for imaging system analysis," *JOSA A,* vol. 24, pp. B25-B41, 2007.

[4] A. Skodras, C. Christopoulos, and T. Ebrahimi, "The JPEG 2000 still image compression standard," *IEEE Signal processing magazine,* vol. 18, pp. 36-58, 2001.

[5] L. Pu, M. W. Marcellin, A. Bilgin, and A. Ashok, "Image compression based on task-specific information," in *2014 IEEE International Conference on Image Processing (ICIP)*, 2014, pp. 4817-4821.

[6] D. Taubman and M. Marcellin, *JPEG2000 Image Compression Fundamentals, Standards and Practice: Image Compression Fundamentals, Standards and Practice* vol. 642: Springer Science & Business Media, 2012.

[7] "Information technology -- JPEG 2000 image coding system: Interactivity tools, APIs and protocols," ed: ISO/IEC 15444-9, 2005.

[8] "Kakadu Software," ed: http://www.kakadusoftware.com, Date last accessed 1-February-2015.

[9] J. Gerdes, U. Schwab, H. Lemke, and H. Stein, "Production of a mouse monoclonal antibody reactive with a human nuclear antigen associated with cell proliferation," *International journal of cancer,* vol. 31, pp. 13-20, 1983.

[10] C. Schlüter, M. Duchrow, C. Wohlenberg, M. Becker, G. Key, H.-D. Flad, and J. Gerdes, "The cell proliferation-associated antigen of antibody Ki-67: a very large, ubiquitous nuclear protein with numerous repeated elements, representing a new kind of cell cycle-maintaining proteins," *The Journal of cell biology,* vol. 123, pp. 513-522, 1993.

[11] R. Yerushalmi, R. Woods, P. M. Ravdin, M. M. Hayes, and K. A. Gelmon, "Ki67 in breast cancer: prognostic and predictive potential," *The lancet oncology,* vol. 11, pp. 174-183, 2010.

[12] M. Dowsett, T. O. Nielsen, R. A'Hern, J. Bartlett, R. C. Coombes, J. Cuzick, M. Ellis, N. L. Henry, J. C. Hugh, and T. Lively, "Assessment of Ki67 in breast cancer: recommendations from the International Ki67 in Breast Cancer working group," *Journal of the National Cancer Institute,* 2011.

[13] X. M. Lopez, O. Debeir, C. Maris, S. Rorive, I. Roland, M. Saerens, I. Salmon, and C. Decaestecker, "Clustering methods applied in the detection of Ki67 hot-spots in whole tumor slide images: An efficient way to characterize heterogeneous tissue-based biomarkers," *Cytometry Part A,* vol. 81, pp. 765-775, 2012.

[14] M. K. K. Niazi, E. Downs-Kelly, and M. N. Gurcan, "Hot spot detection for breast cancer in Ki-67 stained slides: image dependent filtering approach," in *SPIE Medical Imaging*, 2014, pp. 904106-904106-8.

[15] M. K. Khan Niazi, M. M. Yearsley, X. Zhou, W. L. Frankel, and M. N. Gurcan, "Perceptual clustering for automatic hotspot detection from Ki-67-stained neuroendocrine tumour images," *Journal of microscopy,* vol. 256, pp. 213-225, 2014.

[16] M. K. K. Niazi, D. J. Hartman, L. Pantanowitz, and M. N. Gurcan, "Hotspot detection in pancreatic neuroendocrine tumors: Density approximation by α-shape maps," in *SPIE Medical Imaging*, 2016, pp. 97910B-97910B.

[17] M. K. K. Niazi, M. Pennell, C. Elkins, J. Hemminger, M. Jin, S. Kirby, H. Kurt, B. Miller, E. Plocharczyk, and R. Roth, "Entropy based quantification of Ki-67 positive cell images and its evaluation by a reader study," in *SPIE Medical Imaging*, 2013, pp. 86760I-86760I.

[18] J. A. Sethian, *Level set methods and fast marching methods: evolving interfaces in computational geometry, fluid mechanics, computer vision, and materials science* vol. 3: Cambridge university press, 1999.

[19] A. Huisman, A. Looijen, S. M. van den Brink, and P. J. van Diest, "Creation of a fully digital pathology slide archive by high-volume tissue slide scanning," *Human pathology*, vol. 41, pp. 751-757, 2010.
